# Blood Flow Restriction Training for the Intervention of Sarcopenia: Current Stage and Future Perspective

**DOI:** 10.3389/fmed.2022.894996

**Published:** 2022-06-13

**Authors:** Xu-zhi Zhang, Wen-qing Xie, Lin Chen, Guo-dong Xu, Li Wu, Yu-sheng Li, Yu-xiang Wu

**Affiliations:** ^1^Department of Orthopedics, Xiangya Hospital, Central South University, Changsha, China; ^2^Xiangya School of Medicine, Central South University, Changsha, China; ^3^National Clinical Research Center for Geriatric Disorders, Xiangya Hospital, Central South University, Changsha, China; ^4^Department of Health and Kinesiology, School of Physical Education, Jianghan University, Wuhan, China

**Keywords:** sarcopenia, aging, blood flow restriction training, aerobic training, resistance training

## Abstract

Sarcopenia is a geriatric syndrome that is characterized by a progressive and generalized skeletal muscle disorder and can be associated with many comorbidities, including obesity, diabetes, and fracture. Its definitions, given by the AWGS and EWGSOP, are widely used. Sarcopenia is measured by muscle strength, muscle quantity or mass and physical performance. Currently, the importance and urgency of sarcopenia have grown. The application of blood flow restriction (BFR) training has received increased attention in managing sarcopenia. BFR is accomplished using a pneumatic cuff on the proximal aspect of the exercising limb. Two main methods of exercise, aerobic exercise and resistance exercise, have been applied with BFR in treating sarcopenia. Both methods can increase muscle mass and muscle strength to a certain extent. Intricate mechanisms are involved during BFRT. Currently, the presented mechanisms mainly include responses in the blood vessels and related hormones, such as growth factors, tissue hypoxia-related factors and recruitment of muscle fiber as well as muscle satellite cells. These mechanisms contribute to the positive balance of skeletal muscle synthesis, which in turn mitigates sarcopenia. As a more suited and more effective way of treating sarcopenia and its comorbidities, BFRT can serve as an alternative to traditional exercise for people who have marked physical limitations or even show superior outcomes under low loads. However, the possibility of causing stress or muscle damage must be considered. Cuff size, pressure, training load and other variables can affect the outcome of sarcopenia, which must also be considered. Thoroughly studying these factors can help to better determine an ideal BFRT scheme and better manage sarcopenia and its associated comorbidities. As a well-tolerated and novel form of exercise, BFRT offers more potential in treating sarcopenia and involves deeper insights into the function and regulation of skeletal muscle.

## Introduction

Recent years have seen a growing trend toward an aging population. Aging can lead to various diseases, and sarcopenia is one of the prominent geriatric syndromes among them (1). Sarcopenia was proposed by the Asian Working Group for Sarcopenia (AWGS) as an “age-related loss of skeletal muscle mass plus loss of muscle strength and/or reduced physical performance” (2), and it was defined as a progressive and generalized skeletal muscle disorder that is associated with an increased likelihood of adverse outcomes, including falls, fractures, physical disability and mortality, by the European Working Group on Sarcopenia in Older People (EWGSOP) (3). There are many reported risk factors concerning sarcopenia; among them, older age is regarded as the most important, while lifestyle, poor nutritional status and diseases (metabolic diseases and so on) were also independently associated with sarcopenia (2). Muscle strength, muscle mass and physical performance quantity are the three main parameters in the diagnosis of sarcopenia (3). In the guideline proposed by the EWGSOP, muscle strength, mostly measured by grip strength, is regarded as the foremost parameter in predicting adverse outcomes (4). Additionally, physical performance may be used to categorize the severity of sarcopenia (3).

Sarcopenia could be influenced by endogenic and esogenic factors. Genetic factors, such as telomere length (5) and nutritional intake, can impact older adults' pathologies, especially in hospitalized patients (6, 7). The term sarcopenia also encompasses several comorbidities, including cardiometabolic risk factors, notably diabetes, dyslipidemia, osteoporosis (2), respiratory disease (2), cachexia (2), immunosuppression (8), cognitive impairment (9), and sarcopenic obesity (10), because skeletal muscle also acts as a major control center over the metabolic health of the entire body (11).

According to the consensus of the AWGS and EWGSOP, the morbidity of sarcopenia ranges from 3 to 33% (2, 12), and the prevalence rate is as high as 50% in the elderly population over 80 years of age (13). Its substantial tolls, especially to older individuals, are measured according to morbidity, disability (14), and high costs of health care (15). The impact of sarcopenia can be profound and far reaching, causing high personal and social burdens when untreated or poorly treated (16). Notably, sarcopenia is recognized as beginning earlier in life (17). The above findings suggest that sarcopenia requires due consideration and prompt action in being treated, which drives increasing concern toward determining how to effectively manage sarcopenia.

Exercise, including aerobic training and resistance training, can improve the condition of sarcopenia (18). However, there is a growing body of literature that recognizes the importance of blood flow restriction (BFR) training (19). BFR is conducted by applying a pneumatic cuff on the proximal end of the exercising limb. This cuff blocks the return of venous and partially occludes arterial blood flow in the arteriovenous junction, leading to a decrease in blood flow and inducing increased metabolic stress (20).

The issue of BFRT (BFR training) has received considerable critical attention as it increases muscle strength and size at a much lower intensity. The aim of this review is to describe the management of sarcopenia and provide an overview of the underlying mechanisms of BFRT that result in beneficial consequences in treating sarcopenia and rehabilitating skeletal muscles, comparing the advantages and disadvantages of BFRT in curing sarcopenia and discussing the variables that affect the outcomes of therapy and the future applications of BFRT.

## Two Main Methods of BFR Application in Treating Sarcopenia

Currently, researchers agree that BFR shows more effectiveness when combined with exercise training than when used alone. BFR can be coupled with resistance training or aerobic training, which are currently the two main forms in the management of sarcopenia.

### Resistance Training With BFR

BFRT can be coupled with body weight exercise, elastic band resistance exercise, and traditional resistance exercise (21). BFR resistance training, which causes a combination of mechanical and metabolic loads, can prevent the loss of muscle mass and strength caused by increasing age (22).

BFR resistance exercise training is known as “kaatsu training,” which means “training with added pressure.” This method couples low training intensity [~20–50% 1 repetition maximum (RM)] with an external pressure cuff applied to the exercising limb (23), which differs from traditional high load resistance training. In addition, an important difference between high-load and BFRT is that high-load resistance exercise training enhances muscle strength through nervous system adaptation, which is in contrast with BFR exercise training, which increases muscle strength mainly through the mechanism of muscle hypertrophy (24).

BFRT is usually prescribed at low intensity. Because BFRT can efficiently attenuate severe muscle wasting and improve muscle mass and strength, which significantly improves the muscle condition in clinical practice, it is regarded as an effective method to treat sarcopenia (25). Extensive research has also shown that it might serve as a well-tolerated novel intervention that can improve muscle rehabilitation and regeneration to counteract sarcopenia (26).

In a previous report, exercise conducted with BFRT resulted in a significant improvement in the functional capacity of elderly women after 16 weeks (27, 28), demonstrating that BFR-associated lower intensity resistance training can serve as a more effective alternative to low-load resistance training alone and a surrogate for traditional high intensity resistance training. Thus, BFRT can be used as a progressive clinical rehabilitation tool in the process of returning to heavy-load exercise.

### Aerobic Training With BFR

Aerobic training can improve aerobic fitness and arterial function, but it is insufficient to improve muscle mass and strength to counteract the loss of muscle strength that accompanies advancing age (29). However, BFR with an external pressure cuff applied to the upper legs can be combined with aerobic exercise training, such as low-intensity walking or cycling (~20–40% of maximal oxygen consumption). BFR aerobic exercise can not only improve aerobic fitness at a lower intensity but also increase muscle strength and muscle mass (30). Thus, this method can also serve as a novel way of improving muscle ability and can even be applied in the management of sarcopenia.

Other studies have reported that low-intensity BFR walking increases functional ability in older adults, which may be associated with improvements in the overall quality of life and better treatment of sarcopenia (25). Bench-step aerobic exercise with elastic bands is also used in relevant studies to further investigate the mechanism of BFR aerobic training, and significant increases in the vastus lateralis and rectus femoris cross sectional area were observed (25). Overall, the effectiveness of BFRT, when accounting for other variables, requires further exploration.

## The Mechanisms Underlying BFRT in Treating Sarcopenia

There are various causes of sarcopenia and its progression (31). The pathogenesis of sarcopenia includes anorexia (32), inflammation (33), hypogonadism (34), abnormal myokine production (2), hormonal status (2), lack of activity (35), insulin resistance (36), motor neuron loss (37), less active neuromuscular junctions (2), poor blood flow to muscle (38), mitochondrial dysfunction (39), satellite cell senescence (2), and blunting of the anabolic response to food intake (8). Studying the mechanisms of BFRT can offer novel insight into treating sarcopenia.

BFRT, as mentioned above, includes local or partial occlusion of veins and arteries in the arteriovenous junction. BFR may form a partially hypoxic microenvironment. Different cytokines and hormones could participate in the response process. Muscle fiber cells and muscle satellite cells (SGs) are also likely to be involved in BFRT. Overall, responses triggered by BFRT can offset the exacerbated sarcopenia conditions and even cure sarcopenia in future clinical practice. The main mechanisms that underlie BFRT include responses in blood vessels and related hormones, tissue hypoxia-related factors, muscle fiber cells and SGs.

### Responses in Blood Vessels and Related Hormones

Skeletal muscle is highly irrigated by blood vessels (40). In skeletal muscle, endomysial capillaries run parallel to the myofibers (40). The function of muscle microcirculation is to improve muscle contraction that depends on energy substrates (40). The microvasculature can also impact myocyte amino acid availability (11).

Through increasing vascular stress, BFRT triggers hemodynamic responses and thus alters the circulating environment of skeletal muscle. BFRT sessions induced higher systolic and diastolic blood pressure increments (26, 41). It appears that the partial pressure applied to tissue might change the vascular resistance or alter the blood flow output to increase blood pressure. Additionally, ischemic reperfusion following BFRT can induce reactive hyperemic blood flow and increase microvascular filtration capacity (42) in such a way that endothelium-dependent vasodilation during reactive hyperemia is improved (43). A long duration of muscle O_2_ depletion (44) can trigger arteriolar remodeling (45) and even induce angiogenesis through vascular endothelial growth factor (VEGF) (45). The recruited tortuous capillaries contacting myocytes are the nutritive routes essential for muscle perfusion (45). Because BFRT leads to the recruitment of muscle microvasculature (8), reactive hyperemia and hence nutritive flow to the muscle afterward, such as an increase in blood flow and amino acid utilization, can stimulate protein synthesis in muscle directly (46), which can in turn activate the anabolic stimuli of cells (8). All of the mechanisms mentioned above appear to offer a chance to improve the condition of skeletal muscle and thus have beneficial consequences in treating sarcopenia.

However, the mechanisms at the micro level of BFRT on vascular reactivity remain unclear. For example, BFRT does not increase peak forearm blood flow (FBF) or forearm vascular conductance (FVC) in older adults (47), while BFRT did increase peak FVC in the young group; a change in FVC can demonstrate an increased vascular size and increased number of capillaries (42). These results suggest that intriguing mechanisms underlie the observed alternations and open new possibilities for the further exploration of possible reaction channels.

Additionally, because external pressure restricts blood flow, muscle metabolites accumulate in muscle tissue, and these substances can affect muscle responsiveness during BFRT (30) and initiate muscle protein transcription and translation (48). Neuromotor adaptations, combined with tendon and bone adaptations, are also achieved through metabolite accumulation (48, 49). Stimulation of afferent nerves III and IV has been reported, which could in turn lead to the recruitment of type II muscle fibers (50). The molecules induced by metabolite accumulation can include growth hormone (GH), insulin-like growth factor-1 (IGF1), GH-releasing hormone, testosterone (T), and other anabolic- and catabolic-related factors (30). Under pressure conditions, the swelling effect and the secretion of growth hormone and serum testosterone were higher (46).

Hormone imbalances are often associated with sarcopenia (11). A previous study reported that the accumulation of metabolic byproducts leads to the increased secretion of growth hormone (GH) and GH-releasing hormone (51). This effect has been demonstrated, to a large extent, with circulating growth hormone concentrations 290 times higher than baseline reported after acute BFR exercise (52). The increase in growth hormone (GH) and GH-releasing hormone corresponded with the increase in IGF-1. The GH-IGF-1 axis has long been considered a beneficial factor for skeletal muscle growth, while a decrease in the production of IGF-1 is associated with sarcopenia (2). The GH/IGF-1 axis activates the phosphatidylinositol 3 kinase/protein kinase B (AKT) pathway, which increases muscle protein synthesis (MPS) by activating the mammalian target of rapamycin (mTOR) signaling pathway and decreases muscle protein breakdown (MPB) by inactivation of the Forkhead Box O (FOXO) transcription factor (53, 54). Considering these channels, BFRT can greatly improve muscle conditions in patients with sarcopenia. However, some researchers have reported the opposite result. During BFRT and at 10–30 min postexercise, an increase in IGF-1 was observed, while an acute bout of BFRT showed a stagnant level of IGF-1 concentration (23, 55, 56). It is thereby hypothesized that hemoconcentration as a result of plasma volume (PV) changes might be the real cause of the increased level of IGF-1. Therefore, the overall relationship between BFR resistance exercise and the GH-IGF1 axis remains controversial and requires further exploration.

Testosterone (T) is a male hormone that is a steroid hormone and is likely to offset the decline in muscle mass in sarcopenia. Increased testosterone in the BFRT can increase muscle mass and function through its stimulating effect on IGF-1 protein synthesis (57). Only a few reports have observed changes in testosterone, in total or free, following BFR resistance exercise. Madarame et al. reported a postexercise elevation in the total T following BFRT and flexion (55). Thus, the real role that T plays needs further investigation.

Therefore, it is reasonable to conclude that BFRT plays a beneficial role in the management of sarcopenia regarding the microenvironment of skeletal muscle. BFRT helps to improve muscle condition and provide better treatment of sarcopenia. However, more work is required to determine what specific factors play a role and what their contributions are in BFRT.

### Tissue Hypoxia-Related Factors

Hypoxia responses are key elements of BFRT. Energetic and hypoxic stress regulates the muscle growth response in BFRT. The combination of local muscle hypoxia enhances glycolytic cell metabolism, triggers a synthetic hormonal response (57), and activates cellular signaling cascades to increase protein synthesis and inhibit protein degradation (44).

A partially hypoxic environment enhances anaerobic metabolism, increasing muscle biopsy lactate and blood lactate and hence decreasing the pH of muscle cells (30). Usually, lactic acid is produced by fast muscle and transported to the blood by the lactic acid transport carrier MCT4 on the cell membrane (58). Then, it is transported to the vicinity of slow muscle fibers through blood circulation (23, 58). Under pressure, the limited venous blood flow makes it difficult to transport and decompose lactic acid produced by muscles, both during exercise and between exercises (23, 58). This fact can lead to changes in the phenotypes of glycolytic fibers, thus helping to combat sarcopenia. Lactic acid accumulation can also cause muscle cell swelling (59). This effect, in turn, can reduce proteolysis and activation of the mammalian target of rapamycin (mTOR) and mitogen-activated protein kinase (MAPK) pathways (60, 61). Activation of these signaling pathways, especially mTOR, enhances muscle protein synthesis and hence leads to muscle hypertrophy (44). Ultimately, this method provides a new target for curing sarcopenia, which is a beneficial factor in BFRT. A 20% 1-repetition maximum-intensity knee extension exercise with kaatsu can enhance the Akt/mTOR signaling pathway and thigh muscle protein synthesis in young men (62). In addition, in the MAKP pathway, ERK1/2 can phosphorylate Mnk1, which in turn can activate the eIF4E translation initiation factor (46). Presently, ERK1/2 and Mnk1 phosphorylation were reported to increase in BFRT, suggesting that simultaneous activation of mTOR and MAPK signaling pathways may be required for maximum muscle protein synthesis in response to BFRT (63). More studies on different subjects are needed to further investigate the relative mechanisms of the signaling pathways.

Mitochondrial dysfunction in sarcopenia can induce the loss of skeletal muscle mass and neuromuscular junction (64). Anaerobic metabolism can increase mitochondrial function (65), which can preserve and improve the proliferation of muscle cells in sarcopenia. Transient fluxes of several substances, metabolites and nucleotides in skeletal muscle during BFRT may trigger an increase in stimulating transcriptional pathways and signal transduction cascades that ultimately regulate mitochondrial biogenesis programs (66). Sarcopenia is reportedly accompanied by changes in cytoplasmic calcium homeostasis through alterations in dihydropyridine receptors and calcium pump proteins and an increase in cytoplasmic free calcium (67). Sarcoplasmic reticulum Ca^2+^ decreases can be associated with increased cytosolic Ca^2+^, which leads to a decline in muscle strength and quality (67). An increase in mitochondrial production, as important players in buffering cytosolic Ca^2+^ leaked from the sarcoplasmic reticulum, can help cytosolic calcium regulation and buffer its concentration and then enhance both muscle function and strength (67). Training in hypoxia has also shown an angiogenic response in young animals (68), such as an increase in skeletal muscle capillary density (69). From the reports, the pathways in muscle tissues under hypoxia undergo crosstalk with the hormones in the blood vessels, which form an integral system in response to BFRT in sarcopenia.

Heat shock protein is induced by ischemia, hypoxia, and free radicals (70). Increasing HSP72 attenuates atrophy and may play a role in muscle hypertrophy in BFRT (71). However, other researchers have concluded that HSPs do not participate in BFRT as no improvement in HSPs was observed (72, 73). The difference in study subjects may have led to controversial results. Thus, the role of HSPs in BFRT still needs to be verified.

It was previously proposed that hypoxic stress increases the expression of hypoxia-inducible factor (HIF-1) and REDD1, which can inhibit the mTOR pathway (74, 75). However, there are no reports of significant changes in HIF-1 and REDD1 in BFRT (76). Additionally, protein carbonyls and blood glutathione, which are indicators of oxidative stress, were not increased in low-intensity (30% 1RM) BFRT but increased in moderate-resistance exercise (up to 70% 1RM) (77). Therefore, the hypoxia responses and the concrete pathways activated in BFRT need further investigation.

In conclusion, under hypoxia, BFRT activates muscle fiber cells and SGs through several pathways and enhances muscle mass and strength, leading to muscle hypertrophy, which ultimately provides a beneficial condition for managing sarcopenia. During this process, mitochondria play an important role. However, the exact functions of the pathways need verification. More pathways concerning hypoxia might be discovered in the future.

### Recruitment of Muscle Fiber and SGs

On a muscle-specific level, sarcopenia is characterized by a decline in the number of type II muscle fibers and a loss of SGs in type II fibers. A previous study demonstrated that alpha-motor neuron loss can be largely responsible for the loss of muscle mass (78). From this aspect, BFRT can mitigate the effect of sarcopenia through the recruitment of muscle fiber and SGs.

#### Muscle Fiber

In the physiological state, smaller motor units (slow switch type I) are first employed in low-intensity activities, while larger motor units (fast-twitch type II) are then recruited in higher levels of physical training (64). Sarcopenia is accompanied by a reduction in muscle contractile protein, which is associated with decreased strength, mainly due to a reduction in the cross-sectional area of “fast” type II fibers (CSA) (11).

Motor units are also recruited during BFRT (44). Evidence has shown that type II muscle fibers are recruited (30) during BFRT. In BFRT, muscles encounter ischemia, hypoxia, and accumulating metabolites in the tissue, and their responsiveness is influenced by these factors (30). Motor units of more glycolytic fibers are activated to maintain the same level of force generation. This situation may stimulate afferent nerves III and IV, leading to recruitment of type II muscle fibers (44, 79). Recruitment of fast-twitch fibers and increased protein synthesis are accomplished through the target pathway of mTOR (11). BFRT also increases the catabolism of fat and decreases the size of adipose tissue (44), which can combat muscle wasting to cure sarcopenia. BFRT can also provide new insight into treating sarcopenia obesity where muscle tissues are replaced by fat tissues.

Molecular cell signaling involved in muscle hypertrophy responses has been reported in a previous study. Muscle protein synthesis and myogenic gene transcripts, such as proteolytic ligases [muscle-specific ring finger protein-1 (MuRF-1) and Atrogin-1] (46), are upregulated after BFRT (77). The phosphorylation of ribosomal protein S6 kinase beta-1 (S6K1), which is a downstream molecule of the mTOR signaling pathway and regulator of translation initiation and elongation, increases ~3-fold in BFRT (80). A 46% increase in the fractional synthesis rate has been shown following BFR resistance exercise (30). In the related pathway, the phosphorylation of related molecules such as S6K1 and 4E binding protein 1 (4EBP1) is enhanced, leading to translational initiation (60, 61). BFRT leads to an increase in S6K1 and rpS6 phosphorylation, which likely explains the improvement in muscle protein synthesis in BFRT (46, 60). Through the enhanced pathways in muscle cells, BFRT can alter sarcopenia, leading to a positive balance that is beneficial for skeletal muscle conditions.

Neurodegeneration is a key feature of sarcopenia (11). Muscle atrophy and neuromuscular junction (NMJ) remodeling are inevitable in sarcopenia (44). Gamma coactivator 1 alpha (PGC-1α), which improves NMJ structure, is one of the main proteins in preventing muscle destruction and improves muscle conditions in older age with sarcopenia (81). BFR plus low-intensity aerobic exercise can increase the expression of PGC-1α, which in turn likely improves the NMJ structure, and its mandatory proteins may prevent II fiber damage and put them in a better situation (64, 82, 83). PGC-1α reduces the degradation of ubiquitin ligase, facilitates protein degradation, promotes muscle atrophy, and changes proteins in the NMJ synaptic nucleus (84). The unfolded protein response (UPR) is involved in the adaptation of metabolic adjustment and the enhancement of muscle against exercise-related sarcoplasmic reticulum stress (85). In this cycle, PGC-1α starts adaptations in activated muscle by metabolism regulation and myofibril and NMJ genes, which can also contribute to sarcopenia healing (64). BFRT can properly employ both slow- and fast-twitch fibers and leads to a significant increase in PGC-1α in slow muscle and fast muscle to cure sarcopenia (64, 86).

The N-midterminal propeptide of type III procollagen (P3NP) is a subtype of collagen that is located in skeletal muscles and synthesized from the larger procollagen III molecule through the cleavage of N- and C-terminal peptide ends (25) and released into the blood circulation, which is related to the mitigation of muscle loss and sarcopenia. The C-terminal agrin fragment (CAF) shows an increase in the older population and could compromise the integrity of NMJs and lead to muscle atrophy and subsequently decreased muscle strength (25). Researchers have shown that BFRT can improve the functional capacity and circulatory level of P3NP and decrease the circulatory levels of CAF (25). P3NP could be a potential biomarker indicating the curative effect of BFRT on sarcopenia.

On different levels, BFRT shows functions of activation; thus, studying the initial activator may be important. Additionally, muscle fibers demonstrated multifaceted adaptations during BFRT, and further investigations must be performed to verify and discover more potential pathways.

#### SGs

SGs also play an important role in mitigating the negative consequences of sarcopenia and improving muscle function (11). The physiological role of SGs is to provide nuclei to existing myofibers, thereby enhancing or maintaining transcriptional capacity (87). The physiological role of SGs in mediating adaptations to exercise remains a contentious issue. Each nucleus of SGs can only manage a certain volume of cytoplasm, and this so-called karyoplasmatic ratio needs to be maintained (88). Altered regulation of myogenic regulatory factors (MRFs) is involved in the activation and proliferation of quiescent SGs (11). SGs can provide ideal targets for treating sarcopenia through BFRT.

During BFRT, nitric oxide (NO) can activate SGs during exercise by synthesizing hepatocyte growth factor (HGF), which can lead to the proliferation of SGs (89). Then, SGs continue to differentiate and fuse to form new muscle fibers and/or fuse with existing muscle fibers, resulting in muscle fiber hypertrophy and mitigating the decline in muscle mass in sarcopenia. The mTOR pathway also plays a role in the differentiation of SGs (90).

**Figure 1:**
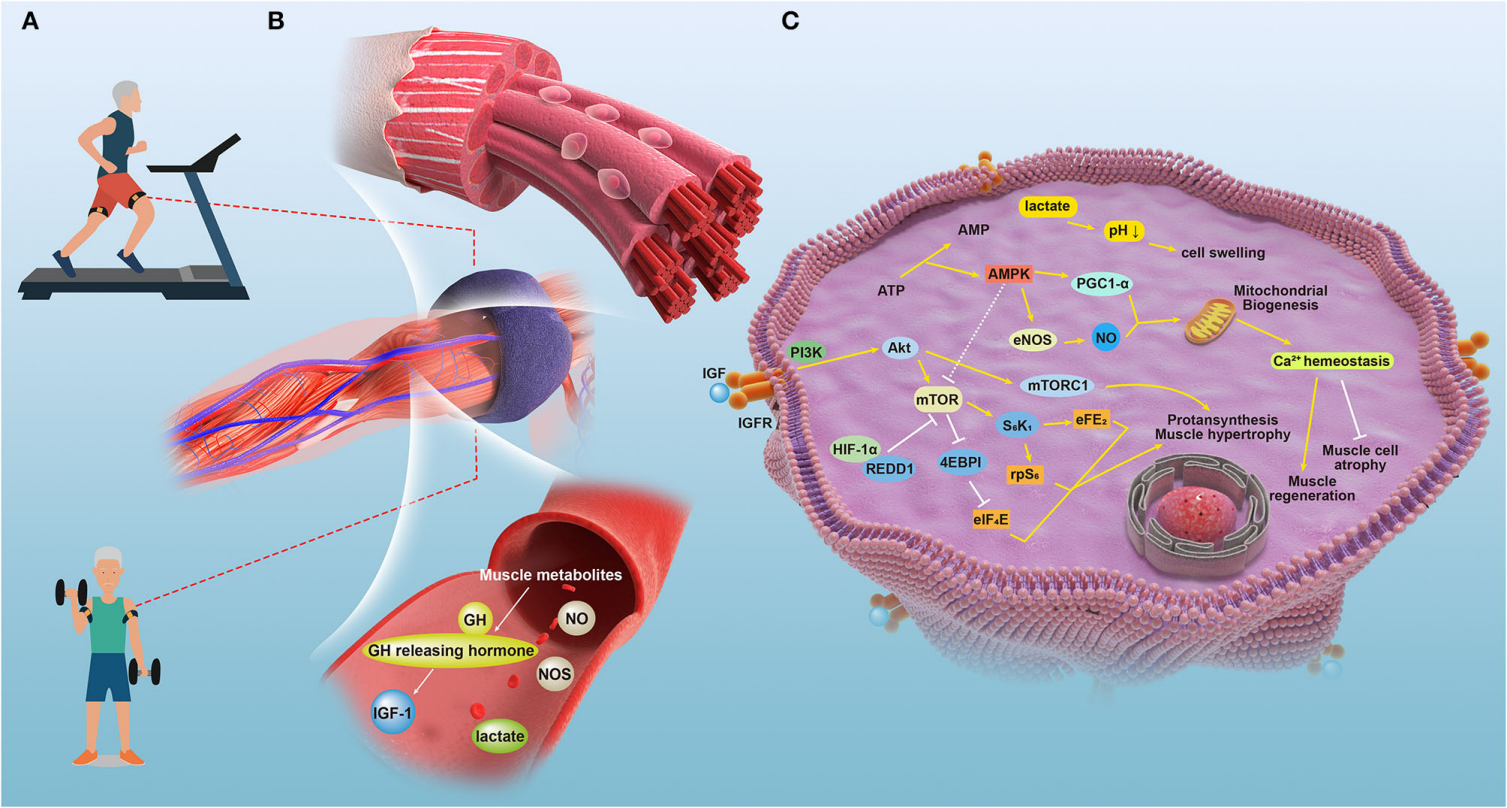
The signaling mechanisms and the underlying crosstalk during BFRT sessions in relative tissues of patients with sarcopenia. **(A)** A depiction of the therapy applying blood flow restriction (BFR) to patient bodies during training sessions. **(B)** Effects induced by the temporal blockage that the local and partial muscles and vasculature underwent when applied with the external pressure of blood flow restriction bands. **(C)** The detailed molecular mechanisms underlying the improvement of physical performance led by BFRT in managing sarcopenia.

Although SGs can be an ideal target for improving muscle rehabilitation and regeneration, more reports have demonstrated that SG reduction is not directly associated with sarcopenia (91). Thus, whether the activation of SGs can improve sarcopenia conditions when sarcopenia and muscle regeneration are independent needs further study and verification. We suggest that more studies be conducted based on the principles of control variables as well as reasonable control groups. The signaling mechanisms and the underlying crosstalk during BFRT sessions are shown in Figure 1.

## Advantages and Disadvantages of BFR in Treating Sarcopenia

As a novel method of exercise training, BFRT can be used as a superior alternative to traditional training to rehabilitate muscle cells, which in turn can possibly mitigate or even cure the negative effect of sarcopenia, especially among older adults (20, 92). However, disadvantages have also been discovered that could induce safety concerns. This circumstance suggests that a novel perspective must be used in investigating BFRT in sarcopenia based on its parameters, namely, muscle strength, muscle quantity or mass and physical performance (3). Deeper insight into BFRT's pros and cons can contribute to finding better treatment methods and more effective and safer management.

### Advantages of BFR

The ESWGOP proposed several standards for an effective method of exercise in treating sarcopenia, which include “to be best suited and most effective for older people,” “enables older people to take more habitual physical activity,” and “alternatives to traditional exercise programs for people who have marked physical limitations” (1, 3). BFRT can fit into such standards.

BFRT can be conducted with a low exercise training load and shows equal or even superior results when compared to traditional high-intensity training. According to the report, two daily BFR sessions combined with low-load resistance training produced significant muscle strength gain and increased muscle mass in only 6 days (93, 94). To achieve the same effect, traditional resistance training requires a longer training duration and a higher training load or volume (95, 96). BFRT can significantly increase thigh muscle size and arterial stiffness (97, 98). In this sense, BFRT is a more effective way to improve muscle strength and physical performance and an alternative to traditional exercise programs.

Sarcopenia is known to be associated with comorbidities, including several underlying diseases (2). BFRT has executable functionality in older individuals with minimal risk of damage (23, 30, 64, 99), and thus, it is suitable for older individuals and those with sarcopenia along with underlying diseases who have marked physical limitations. Patients with sarcopenia who often underwent BFR combined with low-load resistance training did not experience sustained declines in muscle function, exaggerated levels of muscle soreness or increased muscle injury (20, 100, 101), which is beneficial for clinical rehabilitation and elderly populations unsuitable for high loads. Because of its relative ease of implementation and its low load, BFRT enables older individuals to perform more habitual physical activity, which is essential in treating sarcopenia and significantly improves muscle strength, muscle mass and physical performance.

BFRT can increase fibrinolytic activity, thus reducing the risk for blood coagulation (30, 43). Clark et al. observed that tissue plasminogen activator, a fibrinolytic protein, immediately increased following BFRT (27). The accompanying benefits brought by BFRT can offer new insights into treating comorbidities such as cardiometabolic risk factors and diabetes.

Importantly, BFR is accompanied by a “cross-transfer” effect in which endogenous anabolic hormones of blood flow-restricted muscles were observed in non-restricted muscles (82, 102). This finding suggests that BFR can also influence the muscle when it is not under the application of pressure.

### Disadvantages of BFRT

BFR has its limitations. The application of BFR exercise is limited to peripheral muscle groups; thus, core, back, and neck muscles cannot be specifically targeted using this methodology (30, 103).

The other disadvantage of BFRT is that it can lead to a sense of discomfort and even damage to muscle. Higher perceptual ratings and pain during the remaining intervals of sets can also limit the application of BFRT (30, 104). Higher levels of muscle soreness and perceived tiredness have been reported during or following BFRT compared to traditional training (30, 57, 105). The pressure applied to the blood vessel during BFRT is likely the prime cause of the discomfort (98, 106). Myocellular muscle damage and even rhabdomyolysis can occur in response to unaccustomed or excessive BFRT (30, 107). In addition, acute cardiovascular responses to BFRT are also important to consider (30, 108).

Microvascular dysfunction may be the result of reperfusion during blood flow recovery after a period of limitation or ischemia. During reperfusion, there is an acute release of inflammatory molecules and reactive oxides that impair microvascular function. In addition, when blood flow is restored, NO bioavailability (a vasodilator) is reduced, which leads to impaired arterial vasodilation and an increase in pure pressure (43, 107). Repeated reperfusion injury can influence endothelial function. Increased sarcolemma permeability is reported following BFRT, which can be caused by cell damage from the production of reactive oxygen species (30, 109).

BFRT is accompanied by other adverse side effects, such as dizziness, subcutaneous or petechial hemorrhage, drowsiness, numbness, nausea, and itchiness (98, 110). Therefore, we should pay more attention to the safety of BFRT in clinical applications. The advantages and disadvantages reported in sarcopenia, which are mentioned above, are shown in Table 1.

**Table 1:** The advantages and disadvantages reported in sarcopenia.

**Function**	**Advantages**	**Disadvantages**
Muscle capacity	Increased muscle strength corresponds with muscle hypertrophy (24); Gains in physical function led by enhanced muscular power (94); Improvements in muscular endurance led by repetitions (94); Safety method with potential beneficial effects in protecting and augmenting muscle mass and nAChR clustering at the neuromuscular junction (44); More effectiveness in increasing muscle strength compared to low intensity training alone (92)	No nervous system adaptations that result in enhanced muscle strength (24); Lack of training in core, back, and neck muscles (105); Lack of morphological and/or mechanical tendon/bone adaptations (49, 99); Potential myocellular muscle damage and even rhabdomyolysis led by unaccustomed or excessive BFR exercise (107, 108); Less effectiveness compared to heavy-load training (no BFR) (92)
Muscle innervation	Beneficial effect on vascular function of muscle such as arterial compliance and endothelial function (86); Increase in HR and blood pressure (23); Less harmful for the healing of supporting tissues (92)	Concerns of ischemic reperfusion injury (111); Induction of a sympathoexcitatory pressor reflex (104, 110)
Training load	Usage as an effective alternative to low-load training and a surrogate for heavy-load training (100); Potential application as a clinical rehabilitation tool in the process of return to heavy-load exercise (20, 100); Safety in patients early after open cardiac surgery led by well-monitored and stepwise-increased volume (98)	Higher perceptual ratings of perceived exertion and pain during the rest intervals of sets can limit the application (101, 105, 106)
Hemodynamics functions	Increase in fibrinolytic activity to reduce the risk for blood coagulation (30); No increase in fibrinogen/fibrin degradation products and high-sensitive C-reactive protein to indicate affects in hemostatic and inflammatory responses (109)	Concerns of disturbed hemodynamics (111); Complications evoked by the exercise pressor reflex (98)

## Variables That Influence the Effectiveness of BFRT

Several variables can affect the outcome of BFRT; thus, studying these influential variables is necessary for further application of BFRT in managing sarcopenia. Among these variables, cuff size, pressure, training load and intensity, interval between training groups and frequency and the circumference of the limb being exercised are the main variables being discussed (30, 112).

The cuff size, especially the cuff width and the layer of soft tissue situated between the cuff and the vessel, is an important variable for determining a BFRT prescription and could be a limiting factor if not taken into consideration (111). Lower cuff pressures are required to occlude venous blood flow when wider cuffs are used compared to narrow cuffs. However, BFRT performed at supra-systolic blood pressure with wider cuffs can reduce the exercise volume and increase the discomfort compared to narrower cuffs (113). Currently, the consensus is that the narrow cuff has little restriction on movement, good exercise experience, does not easily cause pain and fatigue and is suitable for upper limbs. The wide cuff limits movement, gives a poor exercise experience, can easily cause pain and fatigue but makes it easy to achieve a pressure effect and is suitable for lower limbs (114). The greater the circumference of the limbs, the higher the pressure needed, and a wider cuff could be required to limit the lower extremities (115). A wider cuff is reported to reduce the hemodynamic stress of the BFRT (114).

Pressure is also an important factor in BFR. The cuff width, limb circumference, ankle-brachial index, fat and muscle thickness, arterial stiffness, endothelial function, and blood pressure all influence the BFR pressure (116). The optimal limiting pressure value for BFRT should be high enough to allow venous blood flow to the occluded muscle but low enough to maintain arterial blood flow to the muscle. The optimal limiting pressure value of the BFR varies from person to person and between the upper and lower limbs. Currently, it is generally believed that in low-load resistance exercise, the BFR limiting pressure value can be appropriately selected to be 50–80% of the pressure value required for complete occlusion of arterial flow (114). Previous studies have suggested that to obtain good training results and reduce pain and injury during exercise, the upper limb compression pressure should not be higher than 140 mmHg and the lower limb compression pressure not be higher than 180 mmHg at the beginning of pressure training (107).

The training load and intensity also require further investigation. The training load and intensity were inversely proportional to the number of training repeats, and BFRT had a larger number of repeats per training group than that of the high-load resistance training. In the study of BFR combined with low-load resistance training, the repetition of each training was mostly 45–75 times (56). Of course, more repetitions are not always better; overextending the training time or increasing the number of repetitions can lead to overtraining. The ideal intensity is ~20–50% of one repetition maximum (1RM) (117). It has been reported that 30% 1RM can induce a much higher increase in growth hormone (118).

For the interval between the training groups and frequency, most BFR combined with low-load resistance training uses a relatively short intergroup interval of 30–60 s. Using shorter intergroup intervals is associated with increased metabolic stress to ensure that the body is in a state of incomplete recovery (119), which is the primary mechanism that triggers adaptive muscle responses. Continuous pressure should be maintained during intergroup intervals to further enhance the degree of metabolic stimulation (30). If the pressure is appropriate, the venous flow will be occluded, and the metabolite clearance in the training group will be reduced. Although this accumulation of metabolites undoubtedly affects the performance of the subsequent training, it is likely to be an important mechanism that supports the effectiveness of BFRT (30). In addition, intergroup venous occlusion increases myocyte swelling, which is also thought to play an important role in the adaptive response (86). Studies that implement less frequent BFR resistance exercise over longer training durations also displayed substantial muscular improvement (30).

Postural and impaired muscle histology may also contribute to the response to BFR, which needs further study and exploration. Genetics, total energy intake, macronutrient composition, sex, age and fitness level can influence muscle protein synthesis and breakdown and thus influence the effect of BFRT (30).

Notably, research on the ideal conditions of BFRT was independently conducted in different age and sex groups with different situations and histology, and thus, different factors could interact with one another, causing controversies. Performing systematic research with controlled variables to obtain rigorous results is needed. Additionally, BFRT, when applied in clinical practice, must be careful and cautious in adjusting the variables. The relevant findings on different ways of managing sarcopenia through BFRT are shown in Table 2.

**Table 2:** The management of sarcopenia through BFRT and its research findings.

**References**	**Participants**	**Training load**	**Training repetitions**	**Research type**	**Cuff pressure**	**Effects**	**Mechanisms**
Takano et al. (57)	11 normal healthy adult men, 26–45 years (34 ± 6 years)	20% 1RM	30-30-30-30	Before-after study	160–180 mmHg	Significant rehabilitation in patients was observed	An increase in the circulating insulin-like growth factor-1 (IGF1) at 10–30 min postexercise; A significant increase in GH and VEGF level
Fujita et al. (62)	6 young male subjects	20% 1RM	75	Non-randomized control trial	200 mmHg	Enhanced muscle protein synthesis and improvement of muscle condition were observed.	No increase in circulating IGF1 concentration after180 min following an acute bout; Higher blood lactate, cortisol, and growth hormone levels and activated mTOR.
Drummond et al. (77)	6 young male subjects	20% 1RM	-	Before-after study	-	Skeletal muscle hypertrophy and remodeling were observed. Human muscle gene expression was improved.	No differences at 3 h post-exercise in growth related or proteolytic genes between BFR exercise and non-restricted blow flow exercise; Increase in HIF-1alpha, p21, MyoD, and muscle RING finger 1 (MuRF1) mRNA expression and decrease in REDD1
Fry et al. (60)	7 older male subjects	20% 1RM	75	Cross-over study	120–200 mmHg	Enhanced muscle rehabilitation was observed.	Nine-fold increase in peak GH concentrations in BFR group compared with the Control group. The mTORC1 signaling and MPS were also increased; No changes in selected markers of energetic (AMPK) or hypoxic (HIF-1α, REDD1/2) stress; No significant energetic or hypoxic stress; No influence on the phosphorylation of Akt or FOXO3; No increase in either HSP70 or IL-6 protein expression
Manini et al. (83)	15 subjects (eight men and seven women)	20% 1RM	30-15-15-15	Randomized controlled trail	135–186 mmHg	Improved muscle functions were observed.	Downregulation of the proteolytic genes, FOXO3A, atrogin, and MuRF-1 8 h following BFR exercise; No change in expression of myogenic genes; decrease expression of proteolytic genes associated with muscle remodeling
Cumming et al. (73)	9 healthy volunteers	30% 1RM	-	Before-after study	-	The stress response was more pronounced in type 1 than in type 2 fibers and coincided with low glycogen levels.	Decrease of HSP27 and αB-crystallin levels in the cytosolic and increase of HSP27 and αB-crystallin levels in the cytoskeletal fraction 1 h after exercise; Delayed increase of HSP70 over 48 h
Ganesan et al. (103)	6 young healthy males	50% 1RM	10/set	Before-after study	100 mm Hg	BFR-induced training adaptation and hypertrophyin muscles were observed which was caused by metabolite accumulation in venousblood and subsequent release of circulating factors	Higher [HbR] at the oblique fibers of the vastus medialis muscle and diminished increase in [HbO2]; Higher subjective exertion; Improvement in the delivery of oxygen; Hormonal response led by hypoxia
Farup et al. (96)	healthy young subjects	40% 1RM	-	Before-after study	-	Increased muscle volume by ~12% was observed. Training increased muscle thickness during the immediate 48 h post-exercise.	Transient exercise-induced increases in muscle water content which may be responsible for muscle hypertrophy
Ellefsen et al. (28)	9 untrained women	30% 1RM	-	Non-randomized control trial	-	Shifts in muscle fiber composition in musculus vastus lateralis were observed.	Acute increases in serum levels of human growth hormone; Adaptations in functional, physiological; Activated cell biological parameters
Nielsen et al. (75)	-	30% 1RM	-	Randomized controlled trail	100 mm Hg	Gains in myogenic satellite cell content and muscle hypertrophy were proven.	Observations of signs of tissue inflammation and focal myocellular membrane stress and reorganization
Bjørnsen et al. (93)	17 national level powerlifters	30% 1RM	-	Randomized controlled trail	-	Two blocks of low-load BFR in the front squat exercise resulted in increased quadriceps CSA associated with preferential hypertrophy and myonuclear addition in type 1 fibers of national level powerlifters	Induced selective increases in type I muscle fibers
Lopes et al. (43)	A 91-year-old sedentary man	30% 1RM	-	Case report	-	Muscle mass, handgrip strength and endothelial function were improved.	Increase level of IGF-1; Increase concentrations of TNF-α decrease concentration of IL-6; Improvement of strength, muscle mass, IGF-1, endothelial function, and selected inflammatory markers
Pignanelli et al. (102)	10 healthy, young males	-	25-17-14	Before-after study	-	A greater respiratory capacity increase was observed in muscle endurance.	Type I muscle fiber angiogenesis regardless of the intentional BFR intrinsic changes within mitochondria
Christiansen et al. (112)	Physically trained men	-	-	Before-after study	180 mmHg	Overall muscle improvement was observed.	Increased capacity of pH regulation *via* enhancement of muscle lactate-dependent H^+^ -transport function and blood H^+^ -buffering capacity

## Future Perspectives

Currently, it is generally adopted that the standard protocol of BFR combined with low load resistance training (LL-BFRT) is to perform four sets (1st set: 30 repetitions; 2nd set: 15 repetitions; 3rd set: 15 repetitions and 4th set: 15 repetitions) (95).

The advised BFRT process encompasses a four-step approach: (1) BFR alone during periods of bed rest; (2) BFR combined with low-workload walking exercise; (3) BFR combined with low-load resistance exercise; and (4) LL-BFRT in combination with high-load exercise. Multijoint tasks are regarded as more effective than single joint exercises (95). Thus, LL-BFRT can be used as a progressive clinical rehabilitation tool in the process of returning to heavy-load exercise.

Traditional exercise is sometimes combined with ingredient intake. For example, amino acid levels could be added to a meal to address sarcopenia. Whether protein or amino acid intake during BFRT has a beneficial effect might be an interesting topic for future study. Additionally, a combination of resistance and aerobic exercise may be better in treating sarcopenia (30), and thus, it is worthwhile to study whether these two methods can work synergistically under the application of BFR.

As a well-tolerated exercise and novel intervention that can enhance muscle regeneration to counteract sarcopenia and its comorbidities, BFRT can also be used in the future to manage diseases that are closely related to sarcopenia. In addition, more research should be conducted to explore the mechanisms of BFRT in treating sarcopenia, which can also provide insights about exploring the ideal variables.

## Author Contributions

The first draft of the manuscript was written by X-zZ and W-qX. LC, G-dX, and LW commented on previous versions of the manuscript. Y-sL and Y-xW provided enough scientific suggestions and concrete actions during the revision. All authors read and approved the final version of the manuscript.

## Funding

This work was supported by National Key R&D Program of China (2019YFA0111900), National Natural Science Foundation of China (82071970, 81874030, and 82072506), Provincial Natural Science Foundation of Hunan (2020JJ3060), Provincial Clinical Medical Technology Innovation Project of Hunan (2020SK53709), the Administration of Traditional Chinese Medicine of Hunan Province (2021075), Innovation-Driven Project of Central South University (2020CX045), Wu Jieping Medical Foundation (320.6750.2020-03-14), the Key program of Health Commission of Hunan Province (20201902), Science and Technology Innovation Project of Jianghan University (2021kjzx008), Hunan Yong Talents of Science and Technology (2021RC3025), the Hunan Provincial Innovation Foundation for Postgraduate (CX20210360), and the Independent Exploration and Innovation Project for Postgraduate Students of Central South University (2021zzts1024).

## Conflict of Interest

The authors declare that the research was conducted in the absence of any commercial or financial relationships that could be construed as a potential conflict of interest.

## Publisher's Note

All claims expressed in this article are solely those of the authors and do not necessarily represent those of their affiliated organizations, or those of the publisher, the editors and the reviewers. Any product that may be evaluated in this article, or claim that may be made by its manufacturer, is not guaranteed or endorsed by the publisher.
